# Prescribed Burning and Clear-Cutting Effects on Understory Vegetation in a *Pinus canariensis* Stand (Gran Canaria)

**DOI:** 10.1155/2014/215418

**Published:** 2014-07-24

**Authors:** José Ramón Arévalo, Silvia Fernández-Lugo, Celia García-Domínguez, Agustín Naranjo-Cigala, Federico Grillo, Leonor Calvo

**Affiliations:** ^1^Invasive Species: Interisland Research Group (EIGI), Instituto Universitario de Enfermedades Tropicales y Salud Pública de Canarias (IUETSPC), Universidad de La Laguna (ULL), Avenida Astrofísico Francisco Sánchez s/n, La Laguna 38206, Spain; ^2^Departamento de Geografía, Facultad de Geografía e Historia, Universidad de Las Palmas de Gran Canaria, Las Palmas de Gran Canaria 35003, Spain; ^3^Seguridad y Control de Riesgos, Estructura de Teleformación, Universidad de Las Palmas de Gran Canaria, Las Palmas de Gran Canaria 35003, Spain; ^4^Área de Ecología, Facultad de Ciencias Biológicas y Ambientales, Universidad de León, León 24071, Spain

## Abstract

Prescribed fires are a powerful tool for reducing fire hazards by decreasing amounts of fuel. The main objective is to analyze the effects of prescribed burning on the understory vegetation composition as well as on the soil characteristics of a reforested stand of *Pinus canariensis*. The study attempts to identify the effects of the preburning treatment of cutting understory vegetation on the floristic parameters of the vegetation community. This study was carried out for two years following a prescribed fire in a Canarian pine stand. Cutting and burning treatment affected species composition and increased diversity. Burnt and cut plots were characterized by a diverse array of herbaceous species and by a lower abundance of* Teline microphylla* (endemic legume), although burning apparently induced its germination. Cut treatment was more consistently differentiated from the control plots than burnt treatment. Soil K decreased after both treatments, pH slightly decreased after cutting, while P and Ca increased after fire. From an ecological point of view, prescribed burning is a better management practice than cutting the woody species of the understory. However, long-term studies would be necessary to evaluate the effects of fire intensity, season and frequency in which the prescribed burning is applied.

## 1. Introduction

Historically, fire has played a dominant role in shaping many forest plant communities [1]. Mediterranean-type vegetation is one of the world's major fire-prone biomes [2], with conifer forests among the most flammable ecosystems in the Mediterranean region [3]. In these ecosystems, fire is a crucial process controlling vegetation dynamics and structure [4]. Species have developed different strategies to survive after fire, whether at the individual level, with a thick insulating bark or the ability to resprout from underground parts, or at the population level, with serotinous cones or seeds which are resistant to or stimulated by high temperatures.

Fire regimes have changed as a consequence of human activities and large wildland fires are now more likely to occur. Changing socioenvironmental conditions, such as abandonment of traditional agricultural crops, abandonment of pastoralism, or decreasing exploitation of timber resources, are leading to higher fuel loads and consequently to the increase in the frequency and severity of wildfires [5, 6]. As a result, wildfire prevention measures are necessary and prescribed fires are a powerful management tool towards this goal [1]. In particular, low-intensity prescribed fires are considered useful in some ecosystems to reduce fuel loads by decreasing the amount of low vegetation and small decayed wood [7, 8] without important effects on soil properties [9].

The most recent studies on the effects of prescribed fire in Europe are related to shrublands [4, 10] or grasslands [9], but few deal with pine forests [11]. Studies on vegetation dynamics after wildfires may be used to assess the possible responses of vegetation to prescribed fire under similar conditions. Most studies indicate that although herb and some shrub cover are strongly affected [7], species abundance [12–15] and diversity [16, 17] increase following fire.

Regarding soil properties, prescribed burning has been shown to increase pH and nutrient availability immediately following fire and through the first year [9, 18, 19]. However, these changes usually revert to preburn values after one year. In several studies carried out on the Canarian pine forest, this effect on nutrient content seems to be more persistent [20–25], being possible to detect from 1 up to even 17 years after fire for some nitrogen and phosphorous parameters [22–24]. In contrast, in case of some parameters, such as pH or exchangeable cations, differences might disappear between several months and two years after fire [25, 26].

Canarian forest stands have been subjected to long-term degradation, especially since the European colonization of the islands [27]. Despite being the largest forest community of the islands, only 54% of the Canarian pine forest (60,678 ha) persists nowadays in its natural extension [28]. Restoration programs of the Canarian pine forest have been very common since 1930, existing 15,103 ha of Canarian pine plantation, which require management practices to reach a more natural stage [29]. In this framework, prescribed burning can operate not only as a management tool for fire prevention, but also as a tool for achieving restoration objectives [30].

Little is known about historic fire regimes in the Canary Islands, which makes it difficult to determine which conditions and frequency of burning would be the most appropriate to restore vegetation as well as prevent large wildfires. In the past, wildfire were infrequent but large in extension [20], but after human settlement the frequency of fires increased [33]. At present, natural fire regime has been clearly exceeded, especially in recent decades; thus, most of the Canarian pine forests have been burned in the last 25–30 years, and several stands in that time frame have done so two or three times [34].

If fire management is based on a misunderstanding of plant life history or incorrect historical perceptions, burning could have potentially large effects on forest community diversity [35]. In particular,* Pinus canariensis* is specifically adapted to fire [36], displaying both resistant and resilient strategies, which evidently makes the role of fire during the evolution of this ecosystem important. Moreover, the Canarian pine forest is an ecosystem of particular interest since it has a high percentage of endemic flora and is distinct from other Mediterranean ones. Thus, it is important to study the effect of fire management on this particular ecosystem.

Management of pine forest in Mediterranean countries usually involves cutting the shrub understory to break fuel continuity within the canopy [14]. This practice is also used in the Canary Islands and thus we differentiated between two treatments, understory cutting and subsequent prescribed burning (hereafter “burnt”) and only preburning management (hereafter “cut”). Burning without cutting was not possible due to the danger of canopy torching under the present fuel model. Cutting reduces understory cover and the abundance of vertical fuel ladders. However, due to debris in the field after the cutting treatment, fuel loads remain high and light availability to the soil surface is reduced, which can also affect species composition.

The objective of this study was to analyze the effects of clearing and prescribed fire treatments on the understory community and soil parameters by testing the following main hypotheses: (1) cutting and burning will have different effects on species composition, and different species will be identified as descriptors of each treatment; (2) burning will modify soil parameters more intensively than just cutting, but some of these parameters such as pH or exchangeable cations will recover to pretreatment values in less than two years.

## 2. Methods

### 2.1. Study Site

The study was conducted at the pine forest of Artenara, in Gran Canaria, Canary Islands, Spain (UTM-X 436449, UTM-Y 3099622), which is part of the Protected Landscape of Las Cumbres, under environmental protection by the Canarian Network of Natural Protected Areas [37]. This is a young forest, about 60 years old, which has hardly been managed and never burned since it was planted during restoration management. Pine density is approximately 600 trees per ha with high fuel accumulation. The study site is located between 1400 and 1600 m a.s.l. and faces the prevailing north easterly winds. Mean annual temperature, humidity, and rainfall (for 2006–2008) are 17.7°C, 52.2%, and 500 mm, respectively. The dominant tree species is* Pinus canariensis*, although the area includes other planted exotic pine species such as* P. halepensis* and* P. radiata*. The understory vegetation is dominated by a variety of shrub and herb species, where the most representative species are* Chamaecytisus proliferus, Teline microphylla, *and* Micromeria benthami*. A deep litter layer, with an average thickness of 5.7 cm, typically covers the entire site.

### 2.2. Design of the Experiment

The study site was divided into six plots of between 1.5 and 2 ha each, depending on topographical complexity, where experimental treatments (3 cut and 3 burnt) were applied, and three control plots of approximately 0.5–1 ha each. Control plots were smaller than treated plots to meet management objectives in the largest possible area. From several weeks to a month before burning, the woody understory vegetation of all plots and the lowest branches of the pines were cleared, with the help of chainsaws, except in the controls, as in usual practice to break the vertical continuous fuel ladder before burning. Dead fuel was kept on the ground. This practice allows a homogeneous burning and maintains flame heights of less than 1.5 m due to the spreading of the dead fuel along the surface covering fuel gaps [38]. Three of these six cleared plots were chosen at random and burnt in June 2006 (burnt treatment) while in the other tree plots they were keep as cut treatment. Burning was carried out under specific temperature (18–26°C), humidity (30–70%), and wind (<15 km/h) conditions. Strip fires, a burn method that consists in setting successive parallel strips of fire, and back fires, in which the line of fire is set on the upslope side of the fuel and the fire moves slowly against the wind and slope, were used to achieve ignition. Based on all these parameters and that the canopy only was reached by the flames occasionally, the burning was considered of medium intensity.

Three 100 m^2^ square subplots per treatment plot and one per control plot were located at random. Environmental variables such as rock and litter cover percentage were visually estimated per subplot. Altitude, aspect, and slope were also measured per subplot. All the species in the subplots were listed and their percentage cover was visually estimated and noted on a scale of 1 to 9 (1: trace; 2: <1%; 3: 1-2%; 4: 2–5%; 5: 5–10%; 6: 10–25%; 7: 25–50%; 8: 50–75%; 9: >75%) [31]. Vegetation was sampled six, twelve, eighteen, and twenty-four months after burning.

Four samples of the top 10 cm soil (only organic horizon) were taken per subplot and pooled to obtain a single composite sample before analysis. Soil samples were analyzed following the common standard methods for organic carbon (Walkey-Black method), available phosphorus (Olsen method), potassium, magnesium, calcium, and sodium (Bower method) [39]. Soil pH was analyzed following extraction with deionised water and measured with a pH-meter.

Vegetation and soil sampling were carried out before treatment (spring 2005) and six (winter 2006), twelve (spring 2007; only vegetation), eighteen (winter 2007), and twenty-four (spring 2008) months after burning.

### 2.3. Statistical Analysis

To ensure that there were no significant differences in understory species composition, richness, and diversity before burning and cutting treatment, a distance based permutational MANOVA [40] was fitted, with treatment area (control, burnt, and cut) as fixed factor. The analyses were based on Bray-Curtis distances of the cover data of vascular species and on Euclidian distances of the number of species and diversity prior to treatment (spring 2005). A maximum of 9999 permutations were used to obtain the *P* values (*P* < 0.05). Shannon diversity index: *H* = −Σ*p*_*i*_ln⁡*p*_*i*_, where *p*_*i*_ is the proportion of species *i* relative to the total number of species, was used to characterize species diversity of the pine stand.

In order to test our hypothesis of species composition changes after management practices, distance based permutational-repeated measures MANOVA was fitted, with treatment (control, burnt, and cut) and period (four repeated measures: six, twelve, eighteen, and twenty-four months after treatment) used as fixed factors and the plots as a paired factor. The analyses were based on Bray-Curtis distances of the cover data of understory vascular species. Same procedure was applied to species richness and diversity data but based on Euclidian distances. Significant terms were investigated using a posteriori pairwise comparisons with the PERMANOVA* t*-statistic. A maximum of 9999 permutations were used to obtain the *P* values (*P* < 0.05) and the Monte Carlo correction was applied where necessary.

We performed a similarity percentage (SIMPER) routine [41], using the Bray-Curtis coefficient, to identify both, descriptor species associated with each treatment and those taxa that primarily contribute to average dissimilarity between treatments [42]. This was accompanied by a principal components analysis (PCA) to graphically represent variation in species composition, as it helps to reveal environmental variables not included in the sampling method [43]. We introduced understory species cover data as variables and one datum per treatment per sampling period (the information of the nine subplots that composed the treatment which were pooled into only one, assuming the average of the abundance for the species, based on understory cover data) as samples to reinforce general patterns of species composition depending on the treatment and for a better understanding of the graph. No covariable was introduced for the analysis as none of the environmental variables measured was significantly different between treatments. Primer 6 and PERMANOVA + (PRIMER-E Ltd., Plymouth, UK) were used to perform all statistical procedures, with the exception of the PCA, which was performed using CANOCO for Windows [44].

Finally, to test our second hypothesis we performed one-way distance based permutational ANOVA [40], comparing soil parameters between treatments (fixed factor) for each time period. Analyses were based on Euclidian distances. Significant terms were investigated using a posteriori pairwise comparisons with the PERMANOVA* t*-statistic. A maximum of 9999 permutations were used to obtain the *P* values (*P* < 0.05) in each data set and the Monte Carlo correction was applied where necessary.

## 3. Results

### 3.1. Descriptors and Species Composition

The absence of significant differences in species composition (Pseudo-*F* = 1.18, *P* < 0.05), richness (Pseudo-*F* = 0.92, *P* > 0.05), and diversity (Pseudo-*F* = 0.48, *P* > 0.05) between the treatment areas before being cut and burnt provides assurance that the differences detected between them in the following sampling period are result of management treatments.

**Figure 1:**
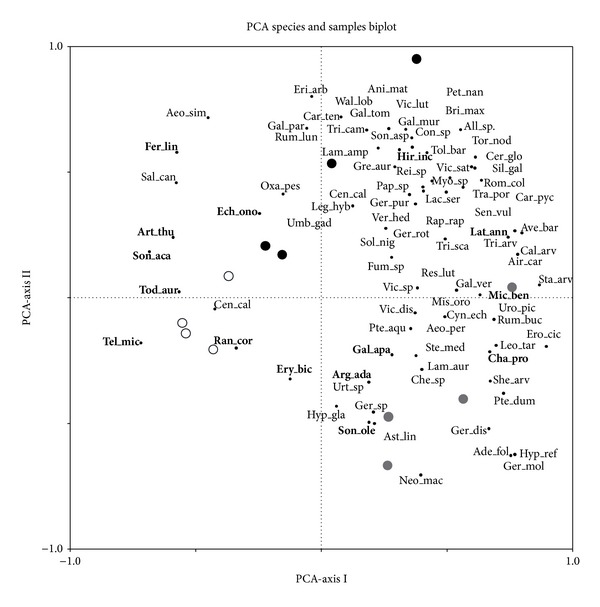
Principal component analysis ordination diagram showing species and samples according to treatment. Species that play consistent roles (SIMPER; mean dissimilarity to SD ratio higher than 1) in determining the dissimilarity between treatments (Table 5) are shown in bold. Species name abbreviations are shown in Table 4. Eigenvalues for the first and second axes were 0.27 and 0.18, respectively. Cumulative percentage variance of species data for both axes was 44.8. Only species with a fit range higher than 10% are shown. Control plots (white circle); burnt plots (black circle); cut plots (brown circle).

We found a total of 107 species belonging to 39 families (12 species were only identified at genus level) and 44% of the species were common to the different treatment areas (Table 4). Cutting and burning treatment affected species composition and diversity (control = 1.91 ± 1.70 SD; cut = 2.88 ± 1.00 SD; burnt = 3.157 ± 0.75 SD) but not species richness (Table 1), which was on average 15.09 ± 9.48 SD. Absence of interaction between treatment and sampling period indicates that changes are maintained over time, as can be appreciated in the PCA graph (Figure 1), where the samples are clearly separated along axis I, with control samples located on the left side of the graph, and treated samples on the right. This separation is mostly due to the abundance of subshrubs and woody species (e.g.,* Teline microphylla*,* Sonchus acaulis*,* Erysimum bicolor*,* Artemisia thuscula*, and* Argyranthemum adauctum*) being more abundant in the control plots, together with some herbaceous species (e.g.,* Ranunculus cortusifolius*,* Sonchus oleraceus*,* Todaroa montana*, and* Galium aparine*). These species play consistent roles (mean dissimilarity to SD ratio higher than 1) in determining the dissimilarity between treatments, especially between control and cut plots more separated in the bidimensional space of the PCA.* Micromeria benthamii *was more abundant in cut than in control plots, while* Hirschfeldia incana* and* Lathyrus annuus* were more abundant in burnt than in control plots (SIMPER; Table 5). Cut and burnt plots were separated along axis II, being characterized mainly by a diverse array of herbaceous species (Figure 1). Although, according to the SIMPER procedure (Table 5) only a few species consistently discriminated between the two treatments. Based on both SIMPER (average dissimilarity between treatments: control versus cut = 72.35, control versus burnt = 61.52, and cut versus burnt = 72.06) and PCA results the cut treatment was more consistently differentiated from the control plots than burnt treatment (Figure 1; Table 5).

Regarding descriptive species responsible for the similarity within treatments, presented using the results from the SIMPER procedure (Table 2), only few species play more or less consistent roles in determining the similarity within treatments.* T. microphylla *was the dominant species in all treatments, having the largest abundance in control plots. As shown above* E. bicolor *and* S. acaulis* were also abundant and consistently present within control plots (Table 2). These three species account for more than 45% of the similarity within control plots. This pattern changed after the treatments, with different results in cut and burnt plots. Cut plots were very variable, without any descriptive species especially abundant and/or consistent within the treated plots. In contrast, burnt plots were more homogeneous, including* T. microphylla *(especially abundant as seedlings) and the herb* Silene vulgaris* as descriptive species (Table 2).

### 3.2. Soil Parameters

The absence of significant differences for any of the soil variables prior to treatment (Table 3) provides assurance that the differences detected between them in the following sampling periods are result of management treatments.

We only detected significant influence of the treatment on some soil parameters six and eighteen months after treatment. Six months after treatment, mean pH values were significantly different between treatments (Pseudo-*F* = 11.66, *P* < 0.00), being lower in cleared plots than in control and burnt ones (Table 3). However, pH recovered to original values 18 months after treatments. P concentrations were significantly different between treatments six (Pseudo-*F* = 6.20, *P* < 0.05) and eighteen months after fire (Pseudo-*F* = 4.89, *P* < 0.05), when P concentrations were higher in burnt plots. Although eighteen months after fire these differences only persist between burnt and cut plots (Table 3). Ca content was significantly lower in cut plots than in burnt ones six months after fire (Pseudo-*F* = 5.47, *P* < 0.05). Finally, K was significantly lower in treated than in control plots six months after management treatment (Pseudo-*F* = 3.95, *P* < 0.05).

## 4. Discussion

Prescribed fire is a useful management tool for preventing large wildfires [1] but can have significant effects on vegetation community diversity [35] and species composition [45], depending on how and where it is applied. Most sources suggest an overall trend of increasing species richness with prescribed fire [13, 15, 16, 45] and a rapid recovery after a low-to-moderate-intensity fire [13, 26]. In most cases, these increases in richness take place during the first to second year after fire and are related to herbaceous pioneer species [15, 45, 46], being linked to canopy opening and higher light availability at soil surface after fire [47]. But studied treatments, understory clearing (cut) and low-intensity prescribed fire (cut), do not imply canopy opening, explaining the absence of significant differences in species richness between treatments (Table 1).

Contrary to species richness, the abundance of understory plants is more sensitive to management [48], leading to the detected changes in species composition (Table 1; Figure 1). The main species leading to differences between treatments was* T. microphylla*, being more abundant in control plots and explaining more than 10% of the dissimilarities found with management treatments. Studies on the soil seed bank suggest that* T. microphylla* germination is stimulated by fire [49], which agree with the results of this research, since despite its lower abundance* T. microphylla *was identified as a descriptor species in burnt plots, where it was especially abundant as seedling but not in cut plots.

Legume shrubs typical of the understory of the pine forest, such as* Adenocarpus foliolosus* and* Chamaecytisus proliferus*, can be classified as pyrophitic species, showing a fast recovery or even increasing their cover after a wildfire [21, 26, 50, 51]. In this study* C. proliferus* was the most abundant in burnt plots, coinciding with recent studies in natural pine forest of the island [26]. Although this species did not consistently discriminate between treatments, it was among the ten species that contributed most to explain the dissimilarities between control and burnt plots (Table 5). In addition, it has been proven that herbaceous legumes play an important role during the early years after fire, showing a typical pioneer strategy of fast growth and propagule production [14, 26, 52]. Consistent with this,* L. annuus *was more abundant in burnt than in control plots, as were* Vicia *spp. and* Trifolium* spp. (Table 5).

Species composition has changed due to management, and two years after the treatment differences remain (Table 1, Figure 1). These differences focus mainly on the higher abundance of some shrubs and perennial herbs in control plots. However attending to the cover classes, with exception of* T. microphylla*, these differences are of small magnitude, remaining in the range of 1-2% of cover. Consequently, we are assuming that only a few more years will be necessary for the shrubs and herbaceous species to increase their abundance in the treated plots, as has been found in other Canarian pine forest stands where only a few years are necessary to recover the prefire species composition [26].

As established in our first hypothesis, both treatments revealed different effects on species composition. Some authors found that, in high-density pine stands, minimal disturbances, such as surface fires, have a low impact on understory composition and production [50, 53]. Fire is an intrinsic element of the Canarian pine forest [54], which can favour regeneration of several understory species [55], which could explain that in the studied pine stand based on SIMPER and PCA results the cut treatment was more consistently differentiated from the control plots than burnt treatment. From this point of view, fire appears as a disturbance for which adaptation is complete in the plant community and offers a natural heterogenic landscape that favours diversity as has been found in other studies, while cutting leads to higher differences with the original forest stand and to lower species diversity (Table 1) [50].

The impact of prescribed fire on soil nutrients is known to be more evident during the first year after fire, increasing the levels of the most important elements (P, N, K, and Ca), while in general after one year, soil nutrient content is more similar between burned and nonburned plots [9, 18, 19]. However, in the Canarian pine forest, fire effects on soil properties seem to be more persistent [20–24]. According to the literature, wildfire effects on nitrogen and phosphorous might be evident 17 years after fire [22–24], changes in pH can be maximum 2-3 years after fire [20], and organic matter might need more than four years to recover prefire values [26]. Nonetheless, long lasting effects of fire on soil nutrients are not a rule in Canarian pine forest, since some studies also reveal that pH and exchangeable cations recover prefire values in less than two years [25, 26], and these short-term effects of fire in soil nutrient have also been found in this study.

As hypothesized, fire modified soil parameters more intensively than just cutting, and treatment effects on soil variables lasted less than two years. Burning effects on soil nutrient can differ in direction and intensity from those caused by clearing treatments [55]. Forest management through clear-cutting can result in a pH decrease [56], as has been found in this study (Table 3), probably as a result of the acidification effects of the pine needles that remained as debris in the field after the cutting treatment. On the other hand, fire impact and ash deposition can increase soil pH [20, 55, 57], which usually returns to prefire values within a short period [19, 20]. However, significant increases might occur only at high temperatures [57], which might have not been generated by the low-intensity prescribed fire experienced in this study, explaining the unexpected absence of fire effects on this variable.

Increases in P and exchangeable cations in the surface soil after a low-intensity prescribed fire have been found as common [9, 19, 58–60], due to the combustion of organic matter and the heating conversion of nutrients from nonavailable to available forms [57, 61, 62]. Although the amount of P was doubled as a result of prescribed fire, this positive effect was not long lasting, as found in other studies [57]. Despite different effects of burning and clearing on soil nutrients [55, 56], detected difference in Ca content between these two treatments might have arisen due to a tendency to lower content of this element in cut plots, already present in initial soil conditions (Table 1). For soil K, a slight decrease, compared to control plots, was found six months after both treatments, probably because this mobile cation might have been lost through leaching [55]. Absence of fire impact on some of the analyzed soil parameters or discrepancies regarding intensity, direction, and durability of these effects with previous studies [22–26] could be the result of differences in fire severity, in burned understory species, and on initial soil nutrient content [57, 60–62].

### 4.1. Management Implications

Based on the obtained results, from an ecological point of view, prescribed fire appears to be a better management practice than simply cutting the woody understory. On the one hand, although none of the management practices resulted in pernicious effects on soil nutrient content, prescribed burning seems to favour a short-term pulse in soil nutrients. On the other hand, two years after treatments fuel reduction aims are accomplished, since dominant shrub species (*T. microphylla*) abundance remains in the range of 1-2% of cover in treated plots, while in control plots average abundance range is between 10 and 25% (Table 2). Nevertheless, after two years, higher species composition similarity between control and burnt plots indicates that, in comparison to clearing, prescribed burning accelerates autosuccession back to prefire vegetation conditions.

It should be noted that the stimulation of* T. microphylla* in burnt plots may be counterproductive if it exceeds pretreatment biomass; nevertheless, longer-term studies are necessary to see how this species develops and how long it takes to attain prefire conditions and exceed them, if this is the case, as well as the evaluation of fire intensity, the season, and the frequency in which the prescribed burning is applied.

Perhaps being an efficient and cheap method of forest fuel removal and fire hazard reduction [63, 64], as well as a tool for increasing recreational values and restoring fire prone ecosystems [32, 65], active management through prescribed burning is very limited and rather unpopular in the Canary Islands and in other regions of Spain [64]. However we believe that, in order to avoid catastrophic fires that endanger property and even human lives (e.g., the recent fires on Gran Canaria in 2013 and La Gomera and Tenerife in 2012), a change in fire management should be implemented, encouraging an active management through prescribed burning, since from an ecological point Canarian pine forest has the ability to recover from fire effects in a short term [26].

## Figures and Tables

**Table 1:** Cutting and burning effects on vegetation in *Pinus canariensis* forest stand (Gran Canaria, Canary Islands). Summary of permutational-repeated measures MANOVA fitted for species composition, richness, and diversity. Results from pairwise *t*-test for sampling period are not included since they just display differences in species composition between sampling seasons (spring versus winter).

	Species composition		Species richness		Species diversity	
PERMANOVA	Pseudo-*F*	*P* (perm)	Pseudo-*F*	*P* (perm)	Pseudo-*F*	*P* (perm)

Treatment	4.78	0.00	2.80	0.07	7.73	0.00
Sampling period	1.59	0.02	1.38	0.24	0.34	0.91
Treatment ∗ sampling period	0.68	0.85	0.27	0.95	0.42	0.88

Pairwise *t*-test	*T*	*P* (perm)	*T*	*P* (perm)	*T*	*P* (perm)

Control-burnt	2.39	0.00	—	—	2.15	0.04
Control-cut	2.08	0.00	—	—	2.10	0.04
Cut-burnt	2.09	0.00	—	—	4.50	0.00

**Table 2:** Cutting and burning effects on species composition in *Pinus canariensis* forest stand (Gran Canaria, Canary Islands). Results of the SIMPER procedure conducted to identify descriptor species associated with each treatment. The taxa which contribute to the similarity within treatment, using a cut-off cumulative percentage of 90%, are listed. Av. Abund.: average abundance of taxa based on a scale of 1 to 9 (1: trace; 2: <1%; 3: 1-2%; 4: 2–5%; 5: 5–10%; 6: 10–25%; 7: 25–50%; 8: 50–75%; 9: >75%) [31]; Av. Sim.: average similarity within treatment subplots of taxa; Sim./SD: mean similarity to standard deviation ratio of taxa; Contrib. %: contribution percentage of the taxa to the similarity; Cum. %: cumulative percentage of the contribution to the similarity.

Species	Av. Abund.	Av. Sim.	Sim./SD	Contrib. %	Cum. %
Control (average similarity 54.59)					
*Teline microphylla *(DC.) P. E. Gibbs & Dingwall	6.25	14.25	2.55	26.11	26.11
*Erysimum bicolor* (Hornem.) DC.	2.67	6.52	3.33	11.94	38.05
*Sonchus acaulis* Dum. Cours.	2.75	4.74	1.36	8.69	46.74
*Silene vulgaris* (Moench) Garcke	2.08	4.55	2.09	8.33	55.07
*Sonchus oleraceus* L.	1.83	4.53	4.67	8.31	63.38
*Ranunculus cortusifolius* Willd.	2.42	4.30	1.36	7.89	71.26
*Todaroa montana* Webb ex Christ	2.08	2.70	0.82	4.94	76.20
*Galium aparine* L.	1.33	2.46	0.97	4.50	80.70
*Artemisia thuscula* Cav.	1.25	1.38	0.64	2.53	83.23
*Hirschfeldia incana* (L.) Lagr.-Foss.	1.00	1.27	0.79	2.33	85.56
*Ferula linkii* Webb	1.17	1.23	0.53	2.26	87.82
*Argyranthemum adauctum *(Link) Humphries	1.00	1.16	0.53	2.13	89.94
*Salvia canariensis* L.	1.08	0.96	0.31	1.75	91.70

Cut (average similarity 26.30)					
*Teline microphylla *(DC.) P. E. Gibbs & Dingwall	1.64	5.12	0.74	19.48	19.48
*Silene vulgaris* (Moench) Garcke	1.53	4.77	0.76	18.13	37.61
*Micromeria benthamii *Webb & Berthel.	1.42	3.95	0.68	15.01	52.63
*Vicia disperma* DC.	0.61	1.84	0.21	7.00	59.63
*Erysimum bicolor* (Hornem.) DC.	1.22	1.23	0.45	4.68	64.31
*Sonchus acaulis* Dum. Cours.	0.89	0.98	0.41	3.72	68.03
*Galium aparine* L.	0.83	0.92	0.44	3.49	71.52
*Todaroa montana* Webb ex Christ	0.92	0.81	0.42	3.08	74.60
*Lathyrus annuus *L.	0.69	0.75	0.38	2.86	77.46
*Ranunculus cortusifolius* Willd.	1.00	0.66	0.39	2.52	79.98
*Sonchus oleraceus* L.	0.69	0.62	0.34	2.35	82.33
*Hirschfeldia incana* (L.) Lagr.-Foss.	0.67	0.52	0.33	1.98	84.31
*Adenocarpus foliolosus* (Aiton) DC.	0.69	0.44	0.16	1.66	85.97
*Stellaria media* (L.) Vill.	0.64	0.42	0.30	1.59	87.56
*Argyranthemum adauctum *(Link) Humphries	0.75	0.40	0.30	1.54	89.09
*Echium onosmifolium *Webb	0.36	0.36	0.17	1.37	90.46

Burnt (average similarity 37.33)					
*Teline microphylla *(DC.) P. E. Gibbs & Dingwall	2.11	7.20	2.39	19.27	19.27
*Silene vulgaris* (Moench) Garcke	2.00	6.16	1.61	16.49	35.76
*Sonchus acaulis* Dum. Cours.	1.56	2.84	0.71	7.61	43.37
*Erysimum bicolor* (Hornem.) DC.	1.58	2.79	0.70	7.48	50.85
*Sonchus oleraceus* L.	1.25	2.65	0.98	7.09	57.94
*Hirschfeldia incana* (L.) Lagr.-Foss.	1.39	2.33	0.82	6.23	64.17
*Ranunculus cortusifolius* Willd.	1.42	2.10	0.67	5.63	69.81
*Lathyrus annuus *L.	0.92	1.30	0.54	3.49	73.30
*Galium aparine* L.	0.78	0.83	0.44	2.24	75.53
*Chamaecytisus proliferus *(L. f.) Link	0.86	0.80	0.38	2.15	77.69
*Todaroa montana* Webb ex Christ	0.94	0.78	0.30	2.10	79.79
*Centranthus calcitrapae* (L.) Dufr.	0.69	0.65	0.37	1.75	81.53
*Andryala pinnatifida* Aiton	0.78	0.65	0.41	1.75	83.28
*Argyranthemum adauctum *(Link) Humphries	0.75	0.60	0.39	1.62	84.90
*Echium onosmifolium *Webb	1.39	0.57	0.28	1.54	86.43
*Papaver rhoeas *L.	0.64	0.49	0.34	1.30	87.73
*Galium parisiense* L.	0.67	0.48	0.33	1.29	89.02
*Stellaria media* (L.) Vill.	0.56	0.43	0.34	1.16	90.18

**Table 3:** Cutting and burning effects on soil in *Pinus canariensis* forest stand (Gran Canaria, Canary Islands). Mean and standard deviation (in brackets) of soil parameters in the control (C), cleared (CT), and burnt (B) plots before treatment and six, eighteen, and twenty-four months after treatment.

	Prefire			6 months			18 months			24 months		
	C	CT	B	C	CT	B	C	CT	B	C	CT	B
pH	6.27 (0.15)	6.29 (0.20)	6.46 (0.18)	6.5 (0.15)^a^	6.18 (0.24)^b^	6.74 (0.23)^a^	6.2 (0.75)	6.28 (0.37)	6.52 (0.32)	6.47 (0.50)	6.44 (0.24)	6.51 (0.31)
% OM	3.47 (0.25)	4.04 (0.67)	3.95 (0.72)	5.03 (2.03)	5.28 (1.42)	4.98 (1.89)	3.87 (1.06)	4.26 (0.82)	4.03 (0.74)	4.27 (0.96)	4.5 (0.60)	4.08 (1.01)
ppm P	12.67 (4.62)	11.56 (5.73)	12.75 (5.75)	10.67 (3.06)^a^	8.89 (7.75)^a^	21.11 (8.25)^b^	14 (5.29)^ab^	10 (5.39)^a^	19.78 (7.97)^b^	6.67 (4.16)	7.56 (7.73)	12.67 (7.42)
Ca	9.33 (1.70)	8.58 (1.50)	9.75 (1.48)	9.53 (2.37)^ab^	8.82 (1.87)^a^	11.82 (1.94)^b^	11.13 (3.79)	9.31 (2.08)	12.02 (3.70)	11 (3.89)	9.38 (1.78)	10.76 (3.24)
Mg	5.53 (1.70)	4.47 (1.50)	4.83 (1.48)	4.47 (2.37)	4.09 (1.87)	4.18 (1.94)	5.53 (3.79)	4.47 (2.08)	5 (3.70)	6.47 (3.89)	4.82 (1.78)	4.84 (3.24)
K	1.37 (1.70)	1.29 (1.50)	1.3 (1.48)	2.3 (2.37)^a^	1.64 (1.87)^b^	1.7 (1.94)^b^	1.9 (3.79)	1.5 (2.08)	1.56 (3.70)	2.00 (3.89)	1.47 (1.78)	1.43 (3.24)
Na	0.87 (0.15)	0.91 (0.20)	0.93 (0.18)	1.37 (0.15)	1.21 (0.24)	1.32 (0.23)	2.5 (0.75)	2.18 (0.37)	2.12 (0.32)	1.83 (0.50)	1.51 (0.24)	1.4 (0.31)

C: control; CT: cut; B: burnt; different letters indicate significant differences (permutational ANOVA, 9999 permutations, *P* < 0.05; pairwise *t*-test, *P* < 0.05).

**Table 4:** Plant species (107 spp.) recorded during the research of the cutting and burning effects on vegetation composition in *Pinus canariensis* forest stand (Gran Canaria, Canary Islands). C: control; CT: cut; B: burnt; Abbrev.: abbreviations used in the PCA; Orig.: origins of the species follow Canarian checklist of wild species [32]; ES: endemic species; EG: endemic genus; II: invasive introduced; MN: maybe native; PI: probably introduced; PN: probably native; SI: secure introduced; SN: secure native.

Family	Species	Abbrev.	Orig.	C	CT	B
Alliaceae	*Allium* sp.	All_sp.			x	x
Apiaceae	*Ferula linkii* Webb	Fer_lin	ES	x	x	x
Apiaceae	*Todaroa montana* Webb ex Christ	Tod_mon	EG	x	x	x
Apiaceae	*Torilis nodosa* (L.) Gaertn.	Tor_nod			x	x
Asteraceae	*Andryala pinnatifida* Aiton	And_pin	ES	x	x	x
Asteraceae	*Argyranthemum adauctum *(Link) Humphries	Arg_ada	ES	x	x	x
Asteraceae	*Artemisia thuscula* Cav.	Art_thu	ES	x	x	x
Asteraceae	*Calendula arvensis *L.	Cal_arv	MN		x	x
Asteraceae	*Carduus pycnocephalus* L.	Car_pyc	MN	x	x	x
Asteraceae	*Carduus tenuiflorus* Curtis	Car_ten	MN			x
Asteraceae	*Centaurea aspera* L.	Cen_asp	MN	x	x	x
Asteraceae	*Conyza* sp.	Con_sp.	SI			x
Asteraceae	*Galactites tomentosus* Moench	Gal_tom	MN	x	x	x
Asteraceae	*Hedypnois rhagadioloides *(L.) F. W. Schmidt	Hed_rha	MN		x	
Asteraceae	*Hypochaeris glabra* L.	Hyp_gla	MN	x	x	x
Asteraceae	*Lactuca serriola* L.	Lac_ser	MN		x	x
Asteraceae	*Leontodon taraxacoides *(Vill.) Mérat	Leo_tar	PI		x	
Asteraceae	*Pericallis webbii *Sch. Bip. & Bolle	Per_web	ES	x	x	x
Asteraceae	*Reichardia tingitana *(L.) Roth	Rei_tin	PN		x	x
Asteraceae	*Senecio teneriffae* Sch. Bip.	Sen_ten	SN		x	
Asteraceae	*Sonchus acaulis* Dum. Cours.	Son_aca	ES	x	x	x
Asteraceae	*Sonchus asper* (L.) A. W. Hill	Son_asp	MN	x	x	x
Asteraceae	*Sonchus oleraceus* L.	Son_ole	MN	x	x	x
Asteraceae	*Sonchus *sp.	Son_sp.		x	x	x
Asteraceae	*Tolpis barbata *(L.) Gaertn.	Tol_bar	MN		x	x
Asteraceae	*Tragopogon porrifolius* L.	Tra_por	MN			x
Asteraceae	*Urospermum picroides* (L.) Scop. ex F. W. Schmidt	Uro_pic	PN		x	
Boraginaceae	*Echium onosmifolium *Webb	Ech_ono	ES	x	x	x
Boraginaceae	*Myosotis* sp.	Myo_sp.		x	x	x
Brassicaceae	*Erysimum bicolor* (Hornem.) DC.	Ery_bic	SN	x	x	x
Brassicaceae	*Hirschfeldia incana* (L.) Lagr.-Foss.	Hir_inc	MN	x	x	x
Brassicaceae	*Raphanus raphanistrum* L.	Rap_rap	SN		x	x
Brassicaceae	*Sisymbrium officinale* (L.) Scop.	Sis_off	MN			x
Campanulaceae	*Legousia hybrida *(L.) Delarbre	Leg_hyb	MN	x	x	x
Campanulaceae	*Wahlenbergia lobelioides *(L. f.) Link	Wah_lob	SN			x
Caryophyllaceae	*Pinus radiata *D. Don	Pin_rad	SI		x	x
Caryophyllaceae	*Silene gallica* L.	Sil_gal	MN		x	x
Caryophyllaceae	*Silene vulgaris* (Moench) Garcke	Sil_vul	MN	x	x	x
Caryophyllaceae	*Stellaria media* (L.) Vill.	Ste_med	PI	x	x	x
Caryophyllaceae	*Petrorhagia nanteuilii *(Burnat) P. W. Ball & Heywood	Pet_nan				x
Caryophyllaceae	*Cerastium glomeratum *Thuill.	Cer_glo	MN	x	x	x
Chenopodiaceae	*Chenopodium *sp.	Che_sp.			x	
Crassulaceae	*Aeonium percarneum *(R. P. Murray) Pit.	Aeo_per	ES	x	x	x
Crassulaceae	*Aeonium simsii *(Sweet) Stearn	Aeo_sim	ES	x		x
Crassulaceae	*Greenovia aurea *(C. Sm. ex Hornem.) Webb & Berthel.	Gre_aur	EG		x	x
Crassulaceae	*Umbilicus gaditanus* Boiss.	Umb_gad	PN	x	x	x
Dipsacaceae	*Pterocephalus dumetorus* (Brouss. ex Willd.) Coult.	Pte_dum	ES		x	x
Ericaceae	*Erica arborea* L.	Eri_arb	SN			x
Fabaceae	*Adenocarpus foliolosus* (Aiton) DC.	Ade_fol	ES		x	
Fabaceae	*Chamaecytisus proliferus *(L. f.) Link	Cha_pro	ES	x	x	x
Fabaceae	*Lathyrus annuus *L.	Lat_ann	PI	x	x	x
Fabaceae	*Teline microphylla *(DC.) P. E. Gibbs & Dingwall	Tel_mic	ES	x	x	x
Fabaceae	*Trifolium arvense* L.	Tri_arv	PN		x	x
Fabaceae	*Trifolium campestre* Schreb. in Sturm	Tri_cam	PN	x	x	x
Fabaceae	*Trifolium scabrum* L.	Tri_sca	PN	x	x	x
Fabaceae	*Trifolium *sp.	Tri_sp.		x	x	x
Fabaceae	*Vicia disperma* DC.	Vic_dis	PN	x	x	x
Fabaceae	*Vicia lutea *L.	Vic_lut	MN	x	x	x
Fabaceae	*Vicia sativa* L.	Vic_sat	MN		x	x
Fabaceae	*Vicia *sp.	Vic_sp.		x	x	x
Fumariaceae	*Fumaria* sp.	Fum_sp.		x	x	x
Geraniaceae	*Erodium cicutarium* (L.) L`Hér. in Aiton	Ero_cic	MN		x	x
Geraniaceae	*Erodium *sp.	Ero_sp.		x	x	x
Geraniaceae	*Geranium dissectum* L.	Ger_dis	MN		x	
Geraniaceae	*Geranium molle *L.	Ger_mol	MN		x	x
Geraniaceae	*Geranium purpureum *Vill.	Ger_pur	MN		x	x
Geraniaceae	*Geranium rotundifolium* L.	Ger_rot	MN		x	x
Geraniaceae	*Geranium *sp.	Ger_sp.		x	x	x
Hypericaceae	*Hypericum reflexum *L. f.	Hyp_ref	ES		x	
Hypolepidaceae	*Pteridium aquilinum *(L.) Kuhn in Kerst.	Pte_aqu	PN	x		
Iridaceae	*Romulea columnae* Sebast. & Mauri	Rom_col	SN		x	x
Lamiaceae	*Lamium amplexicaule* L.	Lam_amp	SI		x	x
Lamiaceae	*Micromeria benthamii *Webb & Berthel.	Mic_ben	ES	x	x	x
Lamiaceae	*Salvia canariensis* L.	Sal_can	ES	x	x	x
Lamiaceae	*Stachys arvensis *(L.) L.	Sta_arv	MN		x	x
Orchidaceae	*Neotinea maculata* (Desf.) Stearn	Neo_mac	PN	x	x	x
Oxalidaceae	*Oxalis pes-caprae* L.	Oxa_pes	II			x
Papaveraceae	*Papaver rhoeas *L.	Pap_rho	II	x	x	x
Pinaceae	*Pinus canariensis* C. Sm. ex DC. in Buch	Pin_can	ES	x	x	x
Pinaceae	*Pinus halepensis* Mill.	Pin_hal	SI			x
Pinaceae	*Polycarpon tetraphyllum *(L.) L.	Pol_tet	MN	x		
Poaceae	*Aira caryophyllea L. *	Air_car	MN		x	x
Poaceae	*Anisantha madritensis* (L.) Nevski	Ani_mad	MN			x
Poaceae	*Anisantha rigida *(Roth) Hyl.	Ani_rig	MN		x	
Poaceae	*Avena barbata* Pott ex Link	Ave_bar	MN		x	x
Poaceae	*Avena fatua* L.	Ave_fat	SN			x
Poaceae	*Briza maxima* L.	Bri_max	MN		x	x
Poaceae	*Cynosurus echinatus* L.	Cyn_ech	MN		x	
Poaceae	*Lamarckia aurea *(L.) Moench	Lam_aur	PN		x	
Poaceae	*Vulpia geniculata* (L.) Link	Vul_gen	MN		x	x
Polygonaceae	*Rumex bucephalophorus* L.	Rum_buc	SN	x	x	x
Polygonaceae	*Rumex lunaria* L.	Rum_lun	ES			x
Primulaceae	A*sterolinon linum-stellatum* (L.) Duby	Ast_lin	PN	x	x	x
Ranunculaceae	*Ranunculus cortusifolius* Willd.	Ran_cor	SN	x	x	x
Resedaceae	*Reseda luteola *L.	Res_lut	PN		x	
Rubiaceae	*Galium aparine* L.	Gal_apa	MN	x	x	x
Rubiaceae	*Galium parisiense* L.	Gal_par	PN	x	x	x
Rubiaceae	*Sherardia arvensis* L.	She_arv	MN		x	x
Rubiaceae	*Galium murale *(L.) All.	Gal_mur	PN			x
Rubiaceae	*Galium *sp.	Gal_sp.		x		
Rubiaceae	*Galium verrucosum* Huds.	Gal_ver	MN		x	
Scrophulariaceae	*Misopates orontiu*m (L.) Raf.	Mis_oro	PN		x	
Scrophulariaceae	*Veronica hederifolia* L.	Ver_hed	MN		x	x
Solanaceae	*Solanum nigrum *L.	Sol_nig	MN		x	x
Urticaceae	*Forsskaolea angustifolia* Retz.	For_ang	ES			x
Urticaceae	*Urtica *sp.	Urt_sp.			x	
Valerianaceae	*Centranthus calcitrapae* (L.) Dufr.	Cen_cal	MN	x	x	x

**Table 5:** Cutting and burning effects on species composition in *Pinus canariensis* forest stand (Gran Canaria, Canary Islands). Results of the SIMPER procedure conducted to discriminate those taxa that primarily contribute to average dissimilarity between treatments. The species which contribute most to the differences between treatments, using a cut-off cumulative percentage of 90%, are listed. Average dissimilarity: control versus cut = 72.35, control versus burnt = 61.52, and cut versus burnt = 72.06. Av. Abund.: average abundance of taxa based on a scale of 1 to 9 (1: trace; 2: <1%; 3: 1-2%; 4: 2–5%; 5: 5–10%; 6: 10–25%; 7: 25–50%; 8: 50–75%; 9: >75%) [31]; Av. Diss.: average dissimilarity between treatments of taxa; Diss./SD: mean dissimilarity to standard deviation ratio of taxa; Contrib. %: contribution percentage of the taxa to the dissimilarity; Cum. %: cumulative percentage of the contribution to the dissimilarity.

Species	Control	Cut	Av. Diss.	Diss./SD	Contrib. %	Cum. %
	Av. Abund.	Av. Abund.				
*Teline microphylla *(DC.) P. E. Gibbs & Dingwall	6.25	1.64	8.95	1.61	12.37	12.37
*Sonchus acaulis* Dum. Cours.	2.75	0.89	4.00	1.34	5.53	17.90
*Erysimum bicolor* (Hornem.) DC.	2.67	1.22	3.76	1.20	5.20	23.10
*Ranunculus cortusifolius* Willd.	2.42	1.00	3.72	1.35	5.14	28.24
*Todaroa montana* Webb ex Christ	2.08	0.92	3.30	1.10	4.56	32.80
*Sonchus oleraceus* L.	1.83	0.69	2.55	1.37	3.52	36.33
*Salvia canariensis* L.	1.08	0.22	2.44	0.71	3.38	39.71
*Chamaecytisus proliferus *(L. f.) Link	0.67	0.72	2.43	0.56	3.36	43.07
*Micromeria benthamii *Webb & Berthel.	0.17	1.42	2.38	1.14	3.28	46.35
*Galium aparine* L.	1.33	0.83	2.10	1.02	2.91	49.26
*Artemisia thuscula* Cav.	1.25	0.06	2.10	1.02	2.90	52.16
*Ferula linkii* Webb	1.17	0.06	2.06	0.91	2.85	55.01
*Silene vulgaris* (Moench) Garcke	2.08	1.53	1.94	0.87	2.69	57.69
*Argyranthemum adauctum *(Link) Humphries	1.00	0.75	1.92	1.01	2.66	60.35
*Hirschfeldia incana* (L.) Lagr.-Foss.	1.00	0.67	1.74	1.13	2.40	62.76
*Stellaria media* (L.) Vill.	0.83	0.64	1.62	0.94	2.25	65.00
*Echium onosmifolium *Webb	0.75	0.36	1.56	0.75	2.15	67.15
*Andryala pinnatifida* Aiton	0.75	0.56	1.48	0.91	2.05	69.20
*Vicia disperma* DC.	0.33	0.61	1.45	0.71	2.01	71.21
*Lathyrus annuus *L.	0.33	0.69	1.22	0.96	1.69	72.90
*Pericallis webbii *Sch. Bip. & Bolle	0.67	0.11	1.18	0.77	1.63	74.53
*Umbilicus gaditanus* Boiss.	0.67	0.19	1.17	0.73	1.61	76.14
*Adenocarpus foliolosus* (Aiton) DC.	0.00	0.69	1.14	0.45	1.58	77.73
A*sterolinon linum-stellatum* (L.) Duby	0.50	0.31	1.09	0.76	1.51	79.24
*Papaver rhoeas *L.	0.58	0.14	1.04	0.81	1.44	80.68
*Centranthus calcitrapae* (L.) Dufr.	0.33	0.36	1.01	0.57	1.40	82.08
*Rumex bucephalophorus* L.	0.25	0.56	0.94	0.63	1.30	83.38
*Hypochaeris glabra* L.	0.25	0.39	0.89	0.60	1.23	84.60
*Carduus pycnocephalus* L.	0.08	0.47	0.70	0.59	0.97	85.57
*Aeonium percarneum *(R. P. Murray) Pit.	0.33	0.19	0.69	0.60	0.96	86.53
*Trifolium scabrum* L.	0.25	0.28	0.67	0.56	0.92	87.45
*Aeonium simsii *(Sweet) Stearn	0.42	0.00	0.66	0.53	0.91	88.37
*Galium parisiense* L.	0.33	0.08	0.64	0.47	0.89	89.26
*Neotinea maculata* (Desf.) Stearn	0.17	0.19	0.63	0.44	0.87	90.13

Species	Control	Burnt	Av. Diss.	Diss./SD	Contrib. %	Cum. %
	Av. Abund.	Av. Abund.				

*Teline microphylla *(DC.) P. E. Gibbs & Dingwall	6.25	2.11	6.75	1.75	10.98	10.98
*Todaroa montana* Webb ex Christ	2.08	0.94	3.08	1.17	5.01	15.99
*Sonchus acaulis* Dum. Cours.	2.75	1.56	3.01	1.26	4.90	20.89
*Ranunculus cortusifolius* Willd.	2.42	1.42	2.73	1.27	4.44	25.33
*Erysimum bicolor* (Hornem.) DC.	2.67	1.58	2.43	1.12	3.95	29.28
*Echium onosmifolium *Webb	0.75	1.39	2.18	0.88	3.55	32.83
*Chamaecytisus proliferus *(L. f.) Link	0.67	0.86	2.09	0.71	3.40	36.23
*Salvia canariensis* L.	1.08	0.33	2.09	0.71	3.40	39.62
*Ferula linkii* Webb	1.17	0.69	1.99	0.95	3.23	42.85
*Artemisia thuscula* Cav.	1.25	0.50	1.83	1.06	2.97	45.83
*Galium aparine* L.	1.33	0.78	1.77	1.11	2.87	48.70
*Hirschfeldia incana* (L.) Lagr.-Foss.	1.00	1.39	1.74	1.16	2.83	51.53
*Argyranthemum adauctum *(Link) Humphries	1.00	0.75	1.56	0.95	2.54	54.07
*Andryala pinnatifida* Aiton	0.75	0.78	1.41	0.98	2.29	56.36
*Stellaria media* (L.) Vill.	0.83	0.56	1.39	0.98	2.25	58.62
*Lathyrus annuus *L.	0.33	0.92	1.37	1.07	2.23	60.85
*Sonchus oleraceus* L.	1.83	1.25	1.31	0.93	2.12	62.97
*Centranthus calcitrapae* (L.) Dufr.	0.33	0.69	1.22	0.78	1.98	64.95
*Papaver rhoeas *L.	0.58	0.64	1.22	0.99	1.98	66.93
*Silene vulgaris* (Moench) Garcke	2.08	2.00	1.18	0.80	1.92	68.85
*Umbilicus gaditanus* Boiss.	0.67	0.44	1.18	0.79	1.92	70.76
*Galium parisiense* L.	0.33	0.67	1.07	0.76	1.75	72.51
*Pericallis webbii *Sch. Bip. & Bolle	0.67	0.03	1.00	0.76	1.63	74.14
*Carduus pycnocephalus* L.	0.08	0.72	0.95	0.71	1.55	75.69
*Vicia disperma* DC.	0.33	0.44	0.95	0.64	1.55	77.24
*Micromeria benthamii *Webb & Berthel.	0.17	0.44	0.84	0.55	1.36	78.60
*Asterolinon linum-stellatum* (L.) Duby	0.50	0.06	0.83	0.67	1.36	79.96
*Stachys arvensis *(L.) L.	0.00	0.53	0.81	0.59	1.31	81.27
*Trifolium scabrum* L.	0.25	0.42	0.73	0.66	1.18	82.45
*Aeonium simsii *(Sweet) Stearn	0.42	0.22	0.72	0.63	1.17	83.62
*Hypochaeris glabra* L.	0.25	0.19	0.62	0.55	1.01	84.64
*Vicia lutea *L.	0.17	0.33	0.58	0.51	0.94	85.58
*Rumex bucephalophorus* L.	0.25	0.22	0.56	0.51	0.90	86.48
*Aeonium percarneum *(R. P. Murray) Pit.	0.33	0.11	0.55	0.57	0.89	87.37
*Trifolium campestre* Schreb. in Sturm	0.08	0.36	0.54	0.55	0.88	88.25
*Geranium rotundifolium* L.	0.00	0.39	0.48	0.56	0.77	89.02
*Erica arborea* L.	0.00	0.31	0.47	0.33	0.77	89.79
*Lactuca serriola* L.	0.00	0.39	0.47	0.50	0.76	90.55

Species	Cut	Burnt	Av. Diss.	Diss./SD	Contrib. %	Cum. %
	Av. Abund.	Av. Abund.				

*Erysimum bicolor* (Hornem.) DC.	1.22	1.58	3.41	0.97	4.73	4.73
*Sonchus acaulis* Dum. Cours.	0.89	1.56	3.18	0.87	4.41	9.14
*Ranunculus cortusifolius* Willd.	1.00	1.42	2.96	1.00	4.10	13.25
*Micromeria benthamii *Webb & Berthel.	1.42	0.44	2.88	0.91	4.00	17.25
*Todaroa montana* Webb ex Christ	0.92	0.94	2.70	0.76	3.75	21.00
*Hirschfeldia incana* (L.) Lagr.-Foss.	0.67	1.39	2.70	1.00	3.75	24.75
*Chamaecytisus proliferus *(L. f.) Link	0.72	0.86	2.55	0.76	3.54	28.28
*Echium onosmifolium *Webb	0.36	1.39	2.49	0.74	3.45	31.73
*Sonchus oleraceus* L.	0.69	1.25	2.47	1.03	3.43	35.16
*Silene vulgaris* (Moench) Garcke	1.53	2.00	2.47	0.76	3.43	38.59
*Teline microphylla *(DC.) P. E. Gibbs & Dingwall	1.64	2.11	2.36	0.77	3.27	41.86
*Lathyrus annuus *L.	0.69	0.92	2.13	0.90	2.95	44.82
*Vicia disperma* DC.	0.61	0.44	1.97	0.64	2.74	47.55
*Galium aparine* L.	0.83	0.78	1.95	0.89	2.70	50.26
*Argyranthemum adauctum *(Link) Humphries	0.75	0.75	1.73	0.88	2.40	52.66
*Andryala pinnatifida* Aiton	0.56	0.78	1.67	0.87	2.32	54.98
*Centranthus calcitrapae* (L.) Dufr.	0.36	0.69	1.55	0.75	2.16	57.14
*Stellaria media* (L.) Vill.	0.64	0.56	1.55	0.81	2.15	59.28
*Carduus pycnocephalus* L.	0.47	0.72	1.50	0.74	2.09	61.37
*Adenocarpus foliolosus* (Aiton) DC.	0.69	0.00	1.40	0.42	1.94	63.31
*Ferula linkii* Webb	0.06	0.69	1.37	0.46	1.90	65.21
*Stachys arvensis *(L.) L.	0.22	0.53	1.37	0.58	1.89	67.11
*Papaver rhoeas *L.	0.14	0.64	1.20	0.66	1.67	68.78
*Galium parisiense* L.	0.08	0.67	1.19	0.63	1.65	70.43
*Geranium rotundifolium* L.	0.19	0.39	1.04	0.54	1.44	71.87
*Trifolium scabrum* L.	0.28	0.42	0.98	0.56	1.36	73.23
*Hypochaeris glabra* L.	0.39	0.19	0.97	0.56	1.35	74.58
*Rumex bucephalophorus* L.	0.56	0.22	0.92	0.59	1.28	75.86
*Umbilicus gaditanus* Boiss.	0.19	0.44	0.90	0.56	1.25	77.11
*Salvia canariensis* L.	0.22	0.33	0.89	0.46	1.23	78.34
*Artemisia thuscula* Cav.	0.06	0.50	0.84	0.60	1.16	79.50
*Lactuca serriola* L.	0.11	0.39	0.78	0.53	1.08	80.57
*Trifolium campestre* Schreb. in Sturm	0.08	0.36	0.76	0.47	1.06	81.63
*Erodium cicutarium* (L.) L'Hér. in Aiton	0.36	0.22	0.68	0.55	0.94	82.57
*Silene gallica* L.	0.25	0.36	0.67	0.57	0.93	83.50
*Erica arborea* L.	0.00	0.31	0.67	0.32	0.92	84.42
*Vicia lutea *L.	0.06	0.33	0.63	0.41	0.87	85.29
*Hypericum reflexum *L. f.	0.22	0.00	0.54	0.33	0.75	86.04
A*sterolinon linum-stellatum* (L.) Duby	0.31	0.06	0.53	0.44	0.74	86.79
*Pterocephalus dumetorus* (Brouss. ex Willd.) Coult.	0.17	0.03	0.49	0.39	0.68	87.47
*Cerastium glomeratum *Thuill.	0.14	0.22	0.49	0.41	0.68	88.15
*Neotinea maculata* (Desf.) Stearn	0.19	0.00	0.48	0.32	0.66	88.81
*Lamium amplexicaule* L.	0.06	0.28	0.44	0.38	0.61	89.42
*Oxalis pes-caprae* L.	0.00	0.17	0.37	0.28	0.52	89.94
*Solanum nigrum *L.	0.06	0.14	0.36	0.31	0.50	90.44
